# Traumatic Insanity

**Published:** 1903-09-26

**Authors:** 


### TRAUMATIC INSANITY.

THERE are several problems in connection with injuries to the head and certain other accidents which are followed by severe mental shock, which it is probable that the general medical practitioner may be called upon to solve. For example he may be asked to say whether in any particular case of such injury the traumatism is likely to be followed by any mental disturbance, or if signs of psychic complications do ensue he may be asked to express an opinion relative to the ultimate prognosis. Again it may fall to his lot to determine, if possible, how far a disturbed mental condition is actually due to preceding trauma and to what extent, if at all, such apparent disturbance is influenced by a prospect of obtaining substantial damages from an individual or a company who may be held responsible for the injury. Such questions as these usually involve much difficulty, and as they are not uncommon the matter is one of considerable importance to the medical practitioner, whose reputation is not unlikely to be at stake. In dealing with the subject, Dr. A. B. Richardson[1] states that the different forms which traumatic insanity may assume are very varied, and, in fact, that almost any clinical form of insanity may have its origin in antecedent trauma. He divides cases into two main classes. In the first he includes those where the mental disturbance either follows immediately after the injury or is associated with symptoms developing from that date. In all such cases there is usually a reasonable proportion between the injury itself and the mental disease that follows. The second class embraces those instances in which there is no reasonable proportion between the injury or shock and the mental disorder which follows. As a general statement, he says, it may be premised that the susceptibility of a given brain organisation to the development of insanity from traumatic causes is, to a great extent, measured by its degree of instability either original and congenital or else acquired through some prejudicial influence either of environment or habits of life. In other words, all brain injuries of a given nature do not produce insanity in equal degree or of like character in all cases. Predisposition is a considerable factor in the causation in these cases, as well as in those of non-traumatic origin. He believes, too, that the general tendency is to overestimate the effect of trauma as a cause of insanity. The friends of an insane person are always ready to magnify the influence of any previous head injury and to minimise the influence of predisposing and other exciting causes, such as heredity or bad habits. As a notable instance of genuine traumatic insanity Dr. Richardson refers to Skae's case where a blow on the head causing a depressed fracture was followed almost immediately by marked changes in disposition and moral characteristics, the patient from being cheerful, of happy disposition, attentive to family and business obligations, becoming the reverse—irritable, morose, immoral and generally unreliable. Trephining, with elevation of the depressed bone, restored the former characteristics and a condition of usual mental health. Epilepsy is a well recognised and not infrequent result of head injury. The mental disorder which results is not different in character from that which follows epilepsy from other causes. Where insanity develops years after a head injury, without evidence of cerebral disturbance in the interim, such as severe or frequent headache, or unusual sensations in the head, Dr. Richardson thinks the attack cannot be ascribed primarily to the trauma. In the second class of cases the injurious effect shown at the time of the accident or soon after is almost wholly psychic or mental in character, the physical injury being slight and inconsequential. In most of these cases there is a marked instability of the nervous system normal to the individual. Sometimes there is a decided hysterical tendency. It may be that the susceptibility is shown only by an introspective tendency and disposition toward melancholy. The symptoms in such cases are nearly always quite characteristic. There is first a moderate shock at the time of the injury. Following this there is a marked tendency to concentrate the attention on the accident and to watch for injurious effects of it. The individual often becomes extremely nervous, cannot sleep well, is unable to concentrate his attention on business matters, and becomes emotional and depressed. When well-marked mental disorder finally develops it is characterised rather by disturbance of the emotions than by fixed delusions. The condition is not purely one of functional neurosis but rather a combination of this with a certain amount of psychic disturbance also.

[1] Amer. Jour. of Insanity, July, 1903.

